# Using a sibling-adoption design to parse genetic and environmental influences on children’s body mass index (BMI)

**DOI:** 10.1371/journal.pone.0236261

**Published:** 2020-07-20

**Authors:** Hannah F. Tavalire, Elizabeth L. Budd, Misaki N. Natsuaki, Jenae M. Neiderhiser, David Reiss, Daniel S. Shaw, Jody M. Ganiban, Leslie D. Leve

**Affiliations:** 1 Prevention Science Institute, University of Oregon, Eugene, Oregon, United States of America; 2 Instutite of Ecology and Evolution, University of Oregon, Eugene, Oregon, United States of America; 3 Counseling Psychology and Human Services Department, College of Education, University of Oregon, Eugene, Oregon, United States of America; 4 Department of Psychology, University of California, Riverside, California, United States of America; 5 Department of Psychology, The Pennsylvania State University, University Park, Pennsylvania, United States of America; 6 Child Study Center, Yale University, New Haven, Connecticut, United States of America; 7 Department of Psychology, University of Pittsburgh, Pittsburgh, Pennsylvania, United States of America; 8 Department of Psychology, George Washington University, Washington, DC, United States of America; University of Hong Kong, CHINA

## Abstract

Dietary and physical activity behaviors formed early in life can increase risk for childhood obesity and have continued negative consequences for lifelong health. Previous research has highlighted the importance of both genetic and environmental (e.g., cultural environment or parental lifestyle) contributions to obesity risk, although these studies typically involve genetically-related individuals residing in the same household, where genetic similarity and rearing environment are inextricably linked. Here we utilize a sibling-adoption design to independently estimate genetic and environmental contributions to obesity risk in childhood and describe how these influences might vary as children age. As part of a prospective adoption study, the current investigation used data from biological siblings reared either apart or together, and nonbiological siblings reared together to estimate the contributions of genetics and environment to body mass indices (BMI) in a large cohort of children (*N* = 711). We used a variance partitioning model to allocate variation in BMI to that which is due to shared genetics, common environment, or unique environment in this cohort during middle childhood and adolescence. We found 63% of the total variance in BMI could be attributed to heritable factors in middle childhood sibling pairs (age 5–11.99; 95% *CI* [0.41,0.85]). Additionally, we observed that common environment explained 31% of variation in BMI in this group (95% *CI* [0.11,0.5]), with unique environment and error explaining the remaining variance. We failed to detect an influence of genetics or common environment in older sibling pairs (12–18) or pairs spanning childhood and adolescence (large sibling age difference), but home type (adoptive versus birth) was an important predictor of BMI in adolescence. The presence of strong common environment effects during childhood suggests that early interventions at the family level in middle childhood could be effective in mitigating obesity risk in later childhood and adolescence.

## Introduction

Childhood obesity is a pervasive public health problem that affects more than 13.7 million children in the United States [1]. The Centers for Disease Control and Prevention (CDC) reports increases in obesity prevalence as children age, estimating that 13.0% of 2- to 5-year olds and 20.6% of 12- to 19-year olds struggle with obesity in the United States [2]. Children with obesity are more likely to experience negative health and psychological outcomes compared with peers of normal weight, such as asthma, high blood pressure, type 2 diabetes, anxiety, and depression [3–5]. Furthermore, childhood obesity often persists into adulthood. A U.S. national, longitudinal study found that 90% of adolescents with obesity continued to struggle with obesity throughout adulthood [6], sustaining their heightened risk for poor health and psychological outcomes.

To better understand and ultimately mitigate the rising incidence of childhood obesity and its life-long challenges, it is important to examine factors predisposing children to obesity. Twin and adoption studies have been paramount in developing our understanding of how a combination of genetic and environmental factors influence obesity risk [7]. However, these studies have yielded varied results about the relative influences attributed to genetics or environment, and often focus on a single age group or adults. Multiple meta-analyses of twin and family studies have identified repeated patterns in genetic and environmental influences on BMI from childhood to adulthood by combining existing cohort data [8–10]. Across twin and family study designs, heritability estimates tend to increase as children age, but begin to decrease into adulthood, after age 18 [8]. Of relevance to the age range of the current sample, heritability estimates range from 0.58 to 0.89 for studies including children aged 5–18 years [8]. Additionally, previous longitudinal studies corroborate meta-analysis findings over time in twin and adoption cohorts, with heritability of BMI increasing over early to middle childhood [11, 12], even despite obesogenic environments thought to solidify suboptimal health behaviors [13]. Furthermore, previous work in adult cohorts of adoptees and their relatives demonstrates that shared environmental effects wain in adulthood, and most of the variance in BMI can be explained by heritable factors [14–16].

While we cannot alter a child’s genetic makeup (in the absence of gene therapies), environmental factors that influence obesity risk represent potentially malleable targets for interventions that may alter gene expression. Environmental factors can include those that are experienced by all children in a household (common environment), and those that are unique to each child in a household (unique environment). Examples of potential common environmental factors associated with childhood obesity include access to physical activity opportunities, green space, and healthy foods, and parental support for healthy eating and exercise behaviors [17–19]. Some of these factors could also serve as unique environmental exposures for children if siblings in a family are exposed to them at different levels, or if siblings spend different amounts of time in the home. Understanding and harnessing the contributing influences of common and unique environmental factors during childhood and adolescence is of critical importance to identifying age-appropriate intervention targets to prevent obesity before it becomes a life-long problem.

Here we build upon previous BMI research using an adoption design where biological siblings are reared together or apart from birth, or are reared from birth with other nonbiological siblings. Previous twin and adoption studies have used traditional ‘ACE’ structural equation modeling approaches developed specifically for twin and family designs [20]. Traditional ACE modeling approaches assume that the environments experienced by individuals sharing a home affect the phenotype of interest equally, regardless of the level of genetic relatedness (the ‘equal environment assumption’), and that results from twin studies are generalizable to broader populations [20]. While these models perform well when statistical assumptions are met, they are challenging to implement under exceedingly complex and varied family structures. Utilizing a statistical approach from non-human quantitative genetics [21] which is amenable to the natural factorial design of relatedness and home sharing combinations found in an adoption dataset, we are able to obtain estimates of genetic and environmental contributions to child BMI that are unconfounded by the effects of co-rearing. Furthermore, our dataset is unique from previous adoption studies of BMI [7, 14–16] because the children were adopted at birth (median age = 2 days; *SD* = 12.45; range = 0–91 days) and measurements used in this study were taken during childhood when the effects of common environment on BMI can be detected. In addition, this project includes the biological siblings of the adoptees who were reared with the birth parents, and additional siblings living in either adoptive or birth home.

The objective of this study was to parse the relative contributions of genetics and environment to the BMI of children using this unique sibling-adoption study design to better understand the etiology of BMI across childhood. Following common practice in twin and family studies, we further decomposed the contribution of environment into those factors common for all members of a household (common environment) and those that are unique to each individual [unique environment; 22] or are due to error. Based on the meta-analyses of twin and family studies in which siblings were co-housed from birth [8–10], we expected the relative contribution of genetic influences to increase with age, and environmental influences to vary by age group, with the BMI of younger children being largely influenced by genetic and *common* environmental factors and the BMI of older children being largely influenced by genetic and *unique* environmental factors.

## Methods

### Human subjects

The study sample was drawn from the Early Growth and Development Study [EGDS; 23] and its companion study, Early Parenting of Children [EPoCh; 24]. Together, these studies encompass a prospective adoption cohort that includes participants who were domestically adopted at birth into a non-biological adoptive home, the adoptees’ biological siblings who remained living with the birth parent(s), and additional nonbiological siblings living in either study home. Participants were included in the current study if: (a) they shared a home with a biological sibling, (b) they had a biological sibling living in a different home, or (c) they shared a home with a nonbiological sibling. Participants were excluded from this sample if: (a) they had missing data on their genetic relationship to their rearing parents or their genetic relationship to other siblings in the study, or were missing BMI data or (b) if their data included extreme outliers for observations of age-corrected BMI (putatively because of reporting error). Using an established approach recommended by the Centers for Disease Control and Prevention, children with BMI z-scores below -4 and above 8 after age-correction were removed [25]. Exclusion of these individuals did not affect the overall sample mean for BMI (*t* = 0.45, *df* = 1769.3, *p* = 0.66).

From an original sample of 897 participants between the ages of 5 and 18, the final sample for the current analysis included 711 children from 414 households with different levels of genetic relatedness (monozygotic twins (12 pairs), full siblings or dizygotic twins (115 pairs), half-siblings (192 pairs), and nonbiological siblings (260 pairs)) residing in the same or different homes, for a total of 579 pairs. The sample was 54% male and children ranged in age from 5 to 18 years old (mean age = 11.30 years; SD = 3.22 years). The mean age difference among all related and unrelated siblings was 3.56 years (SD = 2.35 years); half siblings were an additional 1.06 years apart in age on average compared to full siblings (*t* = -3.84, *df* = 275.3, *p*-value < 0.001). Since heritability has been previously demonstrated to vary across developmental stages [8, 26], we categorized children into middle childhood (age 5.0–11.99) or adolescent groups (age 12.0–18.99) and ran models for these age groups separately (see statistical approach). Child and reporting parent demographic information is described in Tables 1 and 2, respectively. All adult participants (parents/guardians) were provided an information sheet prior to the beginning of an online survey by which data about the child were collected. Consent was implied if the parent or guardian decided to complete the online survey. No data were collected directly from children in this study. The research and consent process received approval by the Institutional Review Board at the University of Oregon.

**Table 1:** Child descriptive statistics by home type.

	Full Sample	Adoptive Home	Birth Home
Mean child Body Mass Index (sd)	19.6 (4.7)	19.2 (4.1)	20.4 (5.8)
Mean child age (sd)	11.3 (3.2)	11.5 (3.0)	11.8 (3.8)
Child sex (% male)	53.6	54.6	51.2
Child race (%)			
Caucasian	58.8	59.8	56.4
Multiracial	21.9	22.0	21.3
African American	17.9	16.4	21.8
Native American	<1.0	<1.0	<1.0
Asian	<1.0	<1.0	-
Native Hawaiian/ Pacific Islander	<1.0	<1.0	-
Unknown	<1.0	1.0	-
Child ethnicity (%)			
Non-Hispanic/unknown	87.1	87.4	86.3
Hispanic	12.9	12.6	13.7
Reared by biological parent (%)	39.2	13.8	99.5
**Total sample size (n)**	**711**	**500**	**211**

**Table 2:** Reporting parent descriptive statistics by home type.

	Full Sample	Adoptive Home	Birth Home
Mean parent age (sd)	47.3 (8.6)	51.0 (6.2)	38.2 (6.6)
Parent sex (% male)	42.5	47.2	31.1
Parent race (%)			
Caucasian	84.0	91.0	67.0
Multiracial	2.8	1.2	6.7
African American	9.0	4.3	20.6
Native American	<1.0	-	1.9
Asian	1.3	1.4	1.0
Native Hawaiian/ Pacific Islander	<1.0	<1.0	-
Unknown	2.1	1.8	2.9
Parent ethnicity (%)			
Non-Hispanic/unknown	95.6	98.0	89.5
Hispanic	4.4	2.0	10.5
**Total sample size (n)**	**720**	**511**	**209**

### Measures

#### BMI

We collected child BMI data from parents using online survey tools. Parents reported their child’s height, weight, age, sex, race, and ethnicity. These data were used to calculate BMI age-corrected z-scores for all children using the US 2000 CDC Growth Charts reference [27]. Parent-report of child height and weight has been demonstrated to yield accurate estimates of BMI in this age group when compared to medical record data [28].

#### Home

Home type was assigned based on the original study design where ‘adoptive’ homes contained the original study (EGDS) adopted children, and ‘birth’ homes were the homes of the birth mothers of the original adoptees. Children raised in the same household were considered to share a home in this analysis. Overall, adoptive families had higher maternal education, higher incomes, and more supportive parenting styles than birth homes [29]; these characteristics are consistent with social and physical environments associated with lower childhood obesity risk [30]. We therefore included home type as a variable in each model in an effort to capture broad differences in these household metrics, not to describe the child’s relatedness to their rearing parents. Relatedness to rearing parents was calculated separately, included as a separate factor in the model, and described the biological relationship of each child to their rearing parent(s) (i.e., whether a child was reared by at least one biological parent). We included biological relationship to the parents in our model to account for any variation in BMI due to passive gene-environment correlation [31], which could otherwise falsely inflate the common environmental variance estimate.

#### Sibling pair genetic relatedness

Genetic relatedness among sibling pairs was computed based on maternal report data collected earlier in the study. Values of relatedness were assigned based on assumed pedigree relationships (monozygotic twins = 1.0; dizygotic twins or full siblings = 0.50; half siblings = 0.25; nonbiological siblings = 0.0). Twin zygosity was determined using the zygosity questionnaire [32].

### Statistical approach

A difference in BMI means by home type (adoptive versus birth) was assessed using a Welch’s two-sample t-test. The complex nature of the study design with many multi-level connections among participants—both in relatedness and common household—provides a unique opportunity to employ a novel statistical approach for the field of human quantitative genetics using existing R software. We used a profiled restricted maximum likelihood model (pREML) in the R package *varComp* [33] to partition variance in BMI using biological and nonbiological siblings reared apart and together. To partition the variance due to shared genetics and shared environment, we assigned pairwise relatedness (monozygotic twins = 1.0, full siblings = 0.50, half siblings = 0.25, unrelated = 0.0) and home sharing (same home = 1, different homes = 0) matrices as the correlation structure of the random effects in each model [34]. This approach is conceptually similar to that used in the traditional ACE twin model, where shared genetics varies categorically across levels of relatedness (e.g., monozygotic versus dizygotic twins) and shared environment often reflects shared household (these approaches are formally compared in [35]). However, the flexibility of the current matrix-based approach allows for the simultaneous consideration of all pairwise relationships and home sharing, including those arising from rare or complex family structures (e.g., a household with a large number of children of many pairwise relatedness combinations). The *varComp* package uses these matrices to assign linear variance-covariance structure and returns the estimated proportions of variance attributable to the structure specified by each matrix of pairwise correlations. As in ACE models, we then used these estimates to partition the total variance observed in BMI into that which can be explained by genetic background (V_A_), common environment (V_C_), and residual- or unique environmental- variance (V_E_; error). Broad-sense heritability (*h*^2^) and the effect of common environment (*c*^2^) were estimated as the proportion of total variance in BMI that can be explained by additive genetic factors (sibling relatedness) or common rearing environment (home), respectively, over the total variance observed in BMI, V_p_. These estimates range from 0 to 1, and express the proportion of the total variance in BMI explained by each factor.

As heritability should be estimated within a population of individuals at the same developmental stage, we separated individuals into two age groups which broadly accounted for differences in both development and expected environment. Children age 5.0–11.99 were included in the ‘middle childhood’ group, while those age 12–18 were classified as ‘adolescent.’ These age ranges are consistent with those used in previous work and reflect long standing, broadly accepted life stage age windows in child research involving populations in the Western world [36, 37]. We hypothesized that children age 5.0–11.99 spend more time in the home, thus experience a more similar common environment, while adolescents have more of a unique environment compared to their siblings. We then ran three variance portioning models including the following subsets: (1) middle childhood sibling pairs (*n* = 260 children across 169 pairs), (2) adolescent sibling pairs (*n* = 200 children across 132 pairs), and (3) sibling pairs that spanned age groups (each pair included one child 5.0–11.99 years old and one child 12–18 years old; *n* = 428 children across 278 pairs). Due to the complex relatedness structure among individuals within a family unit, 177 children (out of total *N* = 711) were included in multiple models because they had multiple siblings across the two age groups. To account for this non-independence among the subsamples, we applied a false discovery rate (FDR) correction to all fixed effect and variance component *p* values and report these adjusted *q* values [38].

The full initial pREML models included sex, race, ethnicity, home type (adoptive or birth), whether a child was reared by at least one biological parent, and the interaction of home type and parent biological status. Because of low representation in some racial groups, race was recoded for subsequent analyses as a factor with three levels: ‘Caucasian’, ‘African American’, or ‘Other’. Pairwise comparisons among racial groups are considered significant below a Bonferroni corrected alpha level of 0.017 (0.05/3). Age was not included as a covariate, as BMI scores were already age-adjusted and sibling pair groupings were based on age. BMI observations were natural log (ln) transformed to meet the assumptions of normality of the model. Model fit of all possible reduced models were compared using Akaike’s Information Criterion (AIC) with the ‘dredge*’* function in the R package *MuMIn* [39]. We then used likelihood ratio testing in the R package *lmtest* [40] to verify that the inclusion of multiple variance components in the final model significantly improved the model fit, or if a more simple correlation structure in the random effect was sufficient [34]. Heritability and *c*^2^ and standard errors and confidence intervals were estimated using the ‘h2GE’ function in the R package *gap* [41]. We then used a permutation test to assess significance of the observed *h*^2^ and *c*^2^ estimates by randomizing BMI across individuals for 999 iterations of the final variance component model to build a unique sampling distribution for each variance component. We then calculated the *p*-value for each component as the proportion of model runs in which each variance component estimate was greater than or equal to the observed value. To determine if *h*^2^ or *c*^2^ estimates were significantly different across age group models, we used a Levene’s test for heterogeneity of variance in the *lawstat* R package [42] to compare model residuals in cases when variance component confidence intervals overlapped. To do this, we reran the final variance component models for each age group containing only that single variance component (e.g., only the relatedness matrix), extracted the residuals from each model, and compared the variance of these residuals using a Levene’s test. If the variance in residuals was not statistically significant among models, we would conclude that the variance component accounted for a similar amount of variance in both models and the resulting *h*^2^ or *c*^2^ estimates were not significantly different. Post-hoc power calculations were run in the *sjstats* package [43]. All statistical analyses were performed in R v3.5.1 [44].

## Results

Age-corrected BMI in this dataset ranged from 11.2 to 47.9, with 55 underweight children (BMI<5^th^ percentile), 441 children of normal weight (5^th^ percentile<BMI<85^th^ percentile), 105 overweight children (85^th^ percentile<BMI<94^th^ percentile), and 110 children with obesity (BMI>95^th^ percentile) for their age. Obesity prevalence in our sample was 15.5%, reflective of the current national prevalence in the United States [2]. Mean BMI differed significantly between the two home types, with children reared in the birth home having an average age-adjusted BMI of 20.43 and children reared in the adoptive home having an average age-adjusted BMI of 19.23 (*t* = -2.71, *df* = 302.8, *p* = 0.007).

The final pREML models for BMI variance component estimation for middle childhood pairs and pairs that spanned age groups included child race and home type, while the final model for adolescent pairs included only home type (birth versus adoptive; Table 3), suggesting that associations of these factors with BMI vary over development. Home type was the only predictor retained in all models, with adolescent children in a birth home having a 14% higher BMI on average than adolescent children in an adoptive home (*p* < 0.001). Birth home was also associated with a 5% increase in BMI in middle childhood pairs (*p* = 0.060) and marginally in sibling pairs that spanned age groups (*p* = 0.053). Differences among the three racial groups remained non-significant after Bonferroni correction (α = 0.017). The final model for middle childhood sibling pairs had a significantly higher log likelihood when both genetic and home sharing matrices were included, versus an identity matrix (default) as the correlation structure of the random effect (ΔLL = 11.40, *df* = 2, χ^2^ = 22.83, *p* < 0.001). Furthermore, a model with a default correlation structure for the random effect had a lower log likelihood than models with either the relatedness (ΔLL = 7.80, *df* = 1, χ^2^ = 14.24, *p* < 0.001) or home-sharing matrix alone (ΔLL = 7.10, *df* = 1, χ^2^ = 14.39, *p* < 0.001). Taken together, these results support the inclusion of both genetic relatedness and common environmental matrices as non-zero variance components in the final model for siblings age 5.0–11.99 [34]. In contrast, there was no evidence that information about relatedness or home-sharing improved the fit of the final model for BMI in adolescent sibling pairs (ΔLL = 0.60, *df* = 1, χ^2^ = 1.26, *p* = 0.26), suggesting that unique environment is a main driver of variation in this group. However, this result should be interpreted cautiously, as a power analysis revealed that a sample size of 350 adolescent children (here, *n* = 200) is required in this model to detect a heritability estimate at least as large as was detected in middle childhood sibling pairs. Additionally, when we compared model residuals between the models of middle childhood and adolescent pairs, the Levene’s test for heterogeneity of variance revealed no significant difference (test statistic = 0.38, *p* = 0.54), suggesting that the genetic variance components did not explain significantly different proportions of variance between these models.

**Table 3:** Heritability of child body mass index (BMI) by age group.

**Middle childhood sibling pairs (n = 260 inds)**	Estimate (SE)^a^	t-value	*q*-value*
Intercept	16.54 (1.02)	131.03	<0.001
Race^b^			
Caucasian vs African American	1.08 (1.04)	2.05	0.087
Caucasian vs Other	1.07 (1.03)	1.95	0.078
Other vs African American	1.00 (1.04)	0.15	1.000
Home Type (Birth Parent Home)^c^	1.05 (1.03)	1.89	0.060
Variance Components^d^			
V_A_	0.025 (0.005)		
V_C_	0.012 (0.004)		
V_E_	0.003 (0.002)		
***h***^***2***^ =	**0.63 (0.11)**	***CI (0*.*41*,*0*.*85)***	**0.024**
***c***^***2***^ =	**0.31 (0.10)**	***CI (0*.*11*,*0*.*51)***	**0.003**
**Adolescent sibling pairs (n = 200 inds)**	Estimate (SE)^a^	t-value	*q*-value*
Intercept	20.73 (1.02)	174.73	<0.001
**Home Type (Birth Parent Home)**^c^	**1.14 (1.04)**	**3.78**	**<0.001**
Variance Components^d^			
V_A_	0.011 (0.010)		
V_C_	0.000 (0.000)		
V_E_	0.033 (0.010)		
*h*^*2*^ =	0.24 (0.23)	***CI (-0*.*21*,*0*.*69)***	0.240
*c*^*2*^ =	0.00 (0.00)	***CI (0*.*00*,*0*.*00)***	1.000
**Sibling pairs across age groups (n = 428 inds)**	Estimate (SE)^a^	t-value	*q*-value*
Intercept	18.41 (1.01)	195.73	<0.001
Race^b^			
Caucasian vs African American	1.06 (1.03)	1.91	0.087
Caucasian vs Other	1.09 (1.03)	3.58	0.078
Other vs African American	0.97 (1.03)	-1.10	0.819
Home Type (Birth Parent Home)^c^	1.05 (1.02)	2.13	0.053
Variance Components^d^			
V_A_	0.005 (0.008)		
V_C_	0.000 (0.000)		
V_E_	0.039 (0.008)		
*h*^*2*^ =	0.12 (0.17)	***CI (-0*.*21*,*0*.*45)***	0.240
*c*^*2*^ =	0.00 (0.00)	***CI (0*.*00*,*0*.*00)***	1.000

*q-values are the false discovery rate (FDR) adjusted p-values based one 3 non-independent models. Uncorrected fixed effect *p*-values were estimated using profiled restricted maximum likelihood models (pREML) while variance component *p*-values were estimated using permutation tests; α = 0.05.

^a^Fixed effect estimates are natural log (ln) back-transformed and represent a multiplicative increase in median BMI.

^b^The reference group for each pairwise comparison among racial groups is listed first. *P*-values for multiple comparisons among racial groups are compared to a Bonferroni-corrected alpha level of 0.017.

^c^Adoptive home is the reference group.

^d^95% confidence intervals are reported for each h^2^ and c^2^ estimate.

In the model for siblings that spanned the two age groups, inclusion of either the genetic relatedness (ΔLL = 0.70, *df* = 1,χ^2^ = 0.514, *p* = 0.474) or the common environmental matrix (ΔLL = 0.00, *df* = 1, χ^2^ = 0.038, *p* = 0.845) did not improve model fit. This model was also underpowered (required *n* = 600 children; here, *n* = 428). We observed the overall variance in sibling pairwise differences in BMI to be higher in the adolescent and across age group datasets than the middle childhood group (‘within-family’ variance; var_MIDDLE_ = 0.040, var_ADOL_ = 0.091, var_ACROSS_ = 0.072). The inverse relationship between within-family variance and power would require larger sample sizes to detect the diminishing heritable and common environmental effects in older sibling pairs.

**Fig 1:**
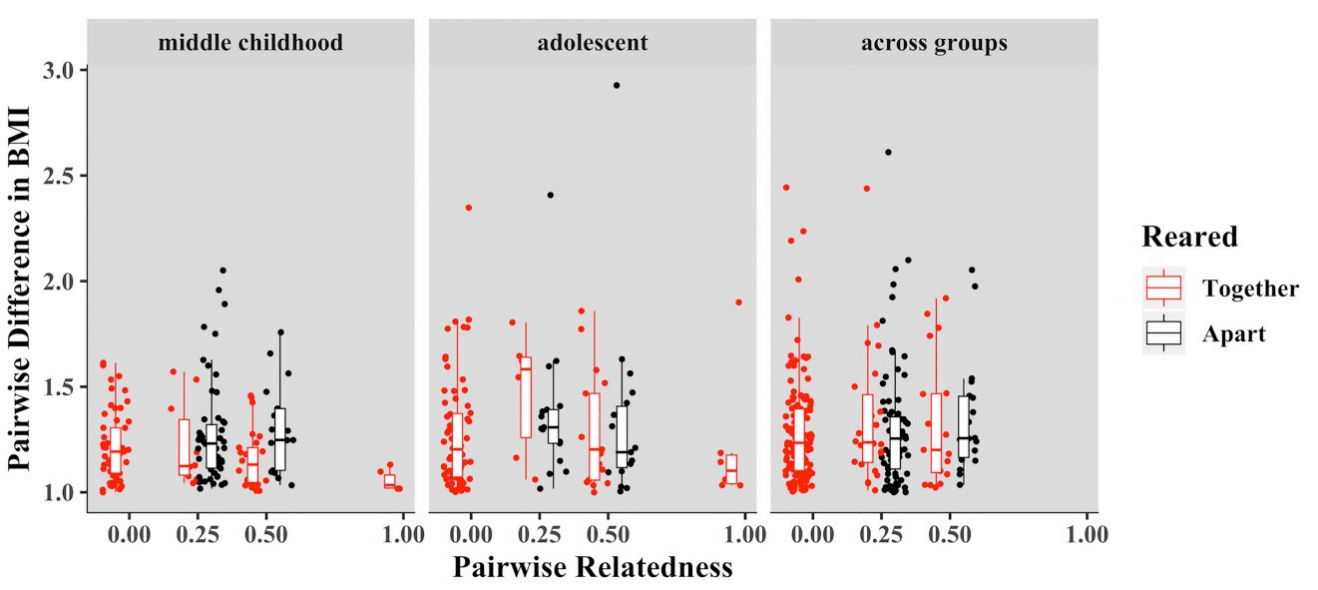
Pairwise difference in BMI by age group. Boxplots depicting the distribution of observed values of pairwise differences in BMI among pairs of varying relatedness and home sharing status within each age group. Each point is a pairwise difference in BMI for a given pair of children in the dataset. In general, differences in BMI were smaller in related children in the middle childhood group, and in those sharing a home environment.

**Fig 2:**
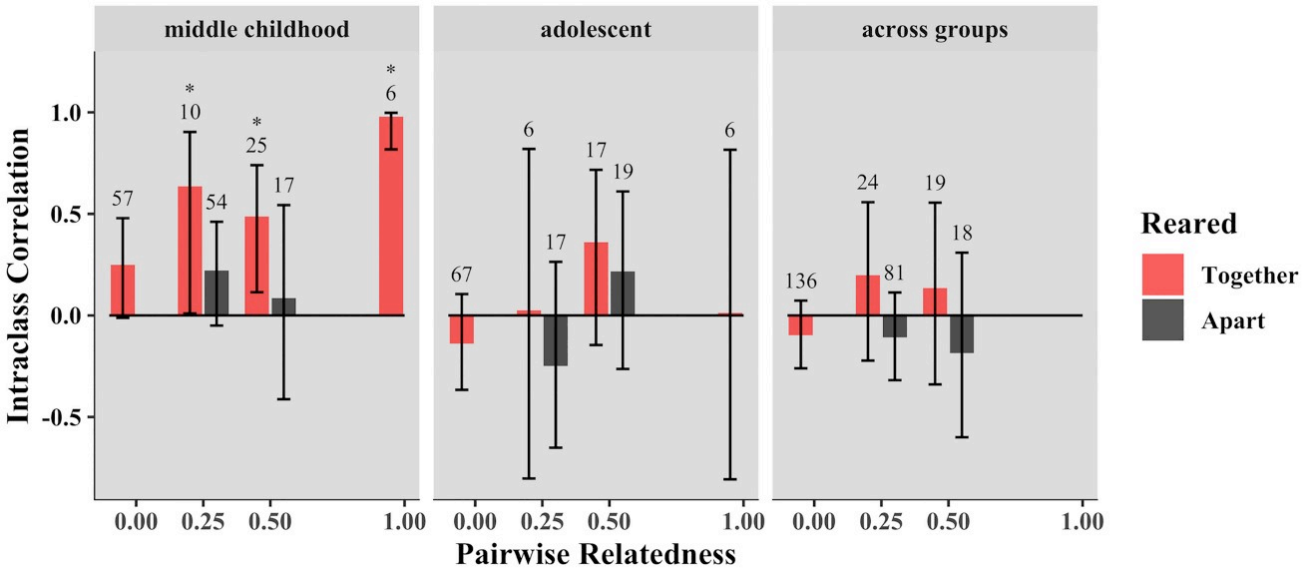
Intraclass correlations for BMI by age group. We observed a general trend of increasing correlation among BMI estimates of related pairs and those sharing a home in middle childhood sibling pairs. Number of pairs of each type are reported above each bar and correlations different from zero are marked with an asterisk (*p* < 0.05).

In middle childhood, we estimated a heritability of 0.63 (SE = 0.11; *p* = 0.008) for BMI, and an effect of common environment of 0.31 (SE = 0.10; *p* < 0.001), with unique environment (error) contributing little to the explainable variance (0.06). Heritable influences and common environment did not significantly contribute to variance in BMI in either adolescent siblings or those sibling pairs that spanned age groups, suggesting that unique environment explained most of the variation in BMI in these age groups (Table 3). Overall, we observed increased similarity in BMI in middle childhood among pairs who were genetically related, especially when reared in the same home (significant intraclass correlations were observed in half siblings (*r* = 0.64), full siblings (r = 0.49), and monozygotic twins (*r* = 0.98) reared together), but this pattern did not persist into adolescence or in sibling pairs that spanned age groups (Figs 1 & 2).

## Discussion

Using a sibling-adoption design in a large cohort, we were able to confirm the unique influence of genetics on child BMI, while simultaneously highlighting the potential importance of the rearing environment for weight status in middle childhood. We obtained significant, non-zero estimates of both heritable (*h*^*2*^) and common environmentally driven (*c*^*2*^) variance components in middle childhood, suggesting important contributions of both genetic and common environmental factors to childhood obesity risk. The obtained heritability estimate of 0.63 falls toward the lower end of the range for other genetically-informed studies that have used more traditional twin methods within similar age groups (e.g., range 0.58–0.89; [8] and is within the range of estimates previously observed in adoption cohorts [7, 15].

Although the heritability of weight status is found to be high in many twin study populations, the influence of common environment is often removed during the model selection process [8]. Prior studies of genetically related children reared together may thus underestimate the role of common and unique environment on child BMI, or may include the effects of non-additive genetic factors in the heritability estimate. Interestingly, this is not the case in previous adoption cohorts, where meta-analysis reveals a variable but sustained influence of common environment until age 13 [7], as was also observed in the middle childhood group here. Further, there is mounting evidence of the influential role of children’s social and physical environments on their learned health behaviors during critical developmental stages [45]. Many of the factors contributing to health behaviors (e.g., socioeconomic status, parental education, food insecurity, access to healthy foods, neighborhood safety, familial stress, and parenting practices; [30, 46] are likely experienced by all children in a home, and would therefore be absorbed into the common environmental variance for a given health outcome. The importance of the home rearing environment is further indicated by the differences seen in BMI between adoptive and birth homes. Aligning with previous work, our results suggest that intervention strategies applied at the level of the family or community context may be effective in mitigating obesity risk in childhood. For example, parenting programs, often involving home visits, are particularly effective at reducing the risk of childhood obesity, whether they target obesity or not [46, 47]. These programs employ ecological or family-systems approaches and primarily aim to foster more secure parent-child relationships and interactions, while simultaneously promoting children’s physical activity and dietary intake at home, at school, and in the community through programs (e.g., youth sports, Safe Routes to School, federally-subsidized meals), policies (e.g., nutrition standards for school foods and beverages, required minimum minutes of Physical Education), and built environmental changes [e.g., installation of crosswalks and sidewalks, improved parks; 18, 19, 48, 49].

Children also learn health behaviors from role modeling and support provided by those in their household. For instance, parental dietary intake and physical activity behaviors associate with a child’s obesity risk throughout early childhood [30, 50]. Additionally, parent physical activity and support for physical activity, whether emotional (e.g., encouragement) or instrumental (e.g., transportation) in nature, are associated with children’s physical activity [30, 50, 51]. Similarly, interventions to promote children’s healthy eating and self-regulation are more effective when parents are highly involved [49], leading to improved psychological wellness (e.g., better self-regulation and reduced stress), engagement in healthy behaviors (e.g., increased physical activity and reduced caloric intake), and years later, improved weight status compared to control groups [52, 53].

Our estimate of common environmental influences on BMI during childhood is higher than those obtained in co-housed twin cohorts of similar ages, where the common environmental variance component is often dropped during model selection [8]. As siblings age, their environments generally become more dissimilar, shifting more of the explainable variance from aspects of the common environment to the unique environment component, leading to instability in variance component estimates in datasets with large age ranges [54]. Here we have accounted for and modeled this variability by splitting sibling pairs into younger and older groups based on established, developmentally and culturally relevant age ranges [36, 37]. Interestingly, in the model for adolescent sibling pairs, we did not detect an effect of common environment, but we did detect a 14% increase in average BMI in adolescents residing in the birth home. Overall, adoptive families had higher maternal education, higher incomes, and more supportive parenting styles than birth homes [29], which is consistent with environments associated with lower childhood obesity risk [30]. This result, taken with the influence of the common environment in middle childhood pairs, suggests that the immediate home environment drives variation in BMI in middle childhood, but broad level differences in education, socioeconomic status, peer contexts, or parenting style may explain variation in BMI in adolescence. These environmental factors may also interact with genetic risk of obesity. For example, higher education is associated with lower obesity levels in individuals at high genetic risk for obesity compared to those individuals of lower education with similar genetic risk [55]. Heterogeneity in influences on BMI during different stages in childhood may offer insight into intervention targets across age classes, focusing more on the immediate home environment in younger children and shifting to the unique contextual environment during adolescence. We were underpowered to detect a significant estimate of heritability in the adolescent sibling pairs model and therefore were unable to replicate previous findings of increasing heritable influences on BMI as children age [7–9]. However, previous twin and family studies do not account for broad differences in households between siblings, since siblings in these studies are co-reared. Here, household type explained significant differences in median BMI between adolescent sibling pairs and siblings that span age groups who were not reared together, suggesting that previous estimates of genetic influences may be inflated by the effects of co-rearing.

In the current study, we introduced a novel statistical approach for estimating variance components for human health outcomes. Building upon foundational work in twin and family designs, we presented a flexible statistical approach amenable to datasets of any relatedness or home sharing structure. This approach provides a complimentary tool to traditional twin model approaches which are limited to datasets containing sibling pairs that share both genes and environments. Conventional twin methods used to study BMI and obesity must assume that all environmental factors affecting these outcomes are equally correlated for identical and fraternal twins, an assumption difficult to prove [the equal environments assumption EEA; 56]. Applying these methods in an adoption cohort- where related siblings are reared in different environments- partially circumvents the EEA.

Though this adoption study provides a novel contribution to our overall understanding of the drivers of variation in BMI in children in a Western population, it is not without limitations. Technical limitations of the design include the use of parent-reported height and weight, the cross-sectional nature of the study, and the geographically and culturally-limited scope of inference. Though parent-reported height and weight have been demonstrated to be accurate in this age group [28], medical records are the gold standard source for assessing child health outcomes. We also detected low power in the adolescent sibling pair and the cross-age models, which could be improved by repeated measures in this cohort. Though this statistical approach is appropriate given the varied family structures in our dataset, methodological limitations of the study design include the possible presence of a violation of the EEA in families with two or more children, and the inability of this sibling-adoption design to detect non-additive genetic contributions to BMI. Future research in this study will include additional characterization of the home environment to add resolution to the home sharing (or home similarity) matrix by including measurable family and community factors previously identified to contribute to obesity risk (e.g., parent-child relationship quality, parent health behaviors, stress, neighborhood walkability). This would allow us to further characterize the main drivers of differences in children’s BMI across birth and adoptive households, that could serve as potential intervention targets. We could also examine how heritable predispositions for physical activity and healthy eating interact with environmental factors, such as parental support and children’s health behaviors and how these influences might vary geographically and culturally.

This sibling-adoption design allowed us to estimate the contributions of genetic background and home environment to child BMI status without confounding genetic relatedness and home sharing between siblings. In addition to replicating the role of genetic variance found in prior studies, we obtained a non-zero estimate of common environmental variance, revealing the importance of common rearing environment in child BMI status in middle childhood. These results support the importance of childhood intervention strategies aimed at modifying the family or contextual rearing environment to mitigate obesity risk in children.

## Supporting information

S1 Dataset BMI and covariate data. This dataset contains measures from deidentified subjects including BMI, sex, race, age, home type, whether each child was reared by a biological parent and age-grouped model inclusion information (n = 711).
(CSV)

S2 Dataset Pairwise relatedness matrix. This dataset includes the pairwise pedigree-based relatedness values for all children in the study (n = 711).
(CSV)

S3 Dataset Pairwise home sharing matrix. This dataset includes the pairwise home sharing values for all children in the study (same home = 1, different home = 0; n = 711).
(CSV)
